# Patent Foramen Ovale: A Fatal Trap

**Published:** 2019-07

**Authors:** Tahereh Davarpasand, Reza Mohseni Badalabadi, Soheil Mansourian, Zahra Rahnamoun

**Affiliations:** *Tehran Heart Center, Tehran University of Medical Sciences, Tehran, Iran.*

**Keywords:** *Foramen ovale, patent*, *Echocardiography, transesophageal*, *Pulmonary embolism*

A 39-year-old man referred to us with a complaint of dyspnea and palpitation of 3 days’ duration. The patient was tachycardic but normotensive with a normal blood oxygen saturation level of about 91%. His electrocardiogram showed a sinus rhythm with an incomplete right bundle branch block. There was no known risk factor for vein thrombosis in his past medical history. Transthoracic and then transesophageal echocardiography revealed a large, hypermobile elongated mass (about 10×1 cm) in the right atrium. The mass was in transit through a large patent foramen ovale (Figure 1, Video 1). There was also severe right ventricular dilation with moderate systolic dysfunction on echocardiography, suggestive of pulmonary thromboembolism (PTE). Consequently, multiple-detector computed tomography angiography was performed to determine mortality risk and help the decision-making regarding the duration of anticoagulation therapy. The angiographic procedure revealed massive bilateral PTE (Figure 2).

The patient was referred for atriotomy and pulmonary embolectomy on cardiopulmonary bypass (Figure 3).

A thrombus in transit is a life-threatening, albeit rare, type of right-heart thrombosis with mortality rates of 80-100% in untreated patients,^1^ necessitating urgent assessment and treatment. A thrombus in transit can result in catastrophic systemic embolism in a patient with PTE; therefore, taking heed of this issue in the presence of a right atrial mass is of great therapeutic significance. Meticulous imaging modalities in such patients are mandatory to prove the existence of a patent foramen ovale with a view to deciding on an emergent individualized therapeutic management of the patient’s condition. 

**Figure 1:**
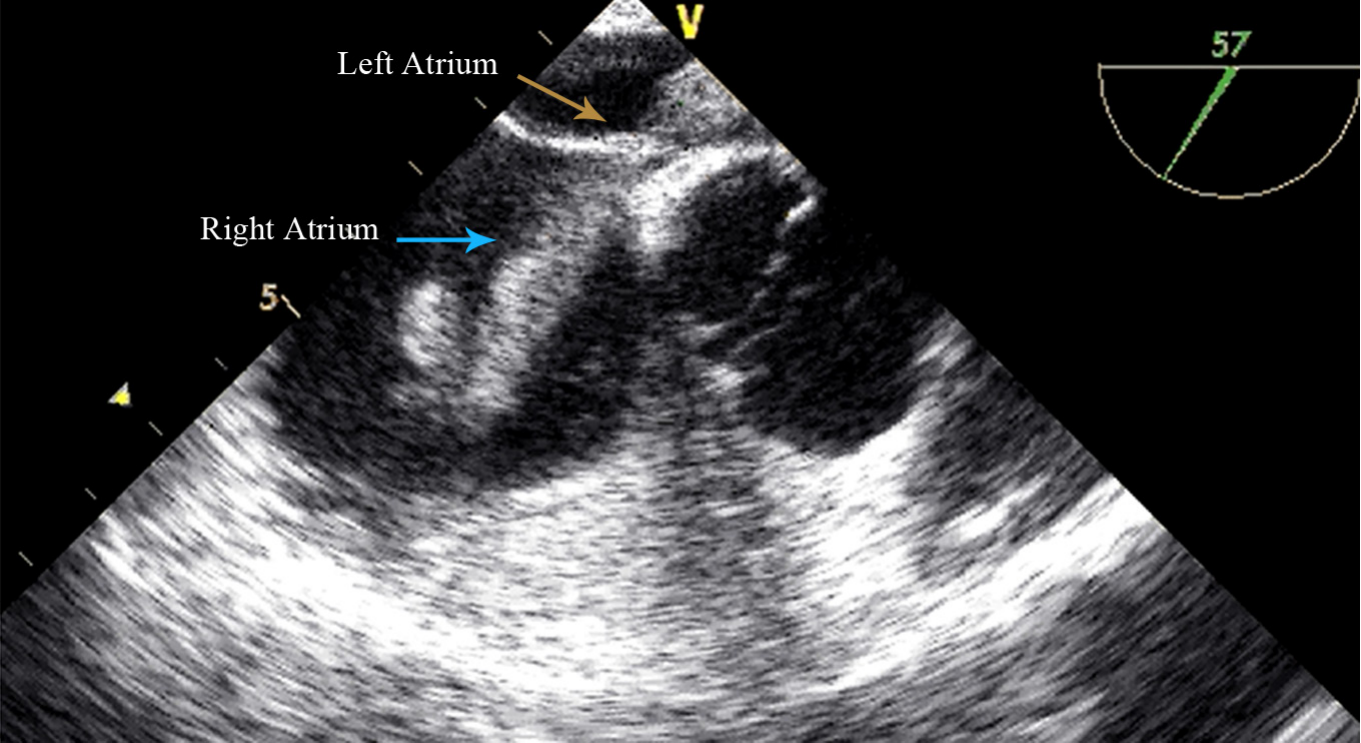
Transesophageal echocardiography (mid-esophageal short-axis view), showing a large hypermobile mass (blue arrow) in transit in a patent foramen ovale (orange arrow)

**Figure 2:**
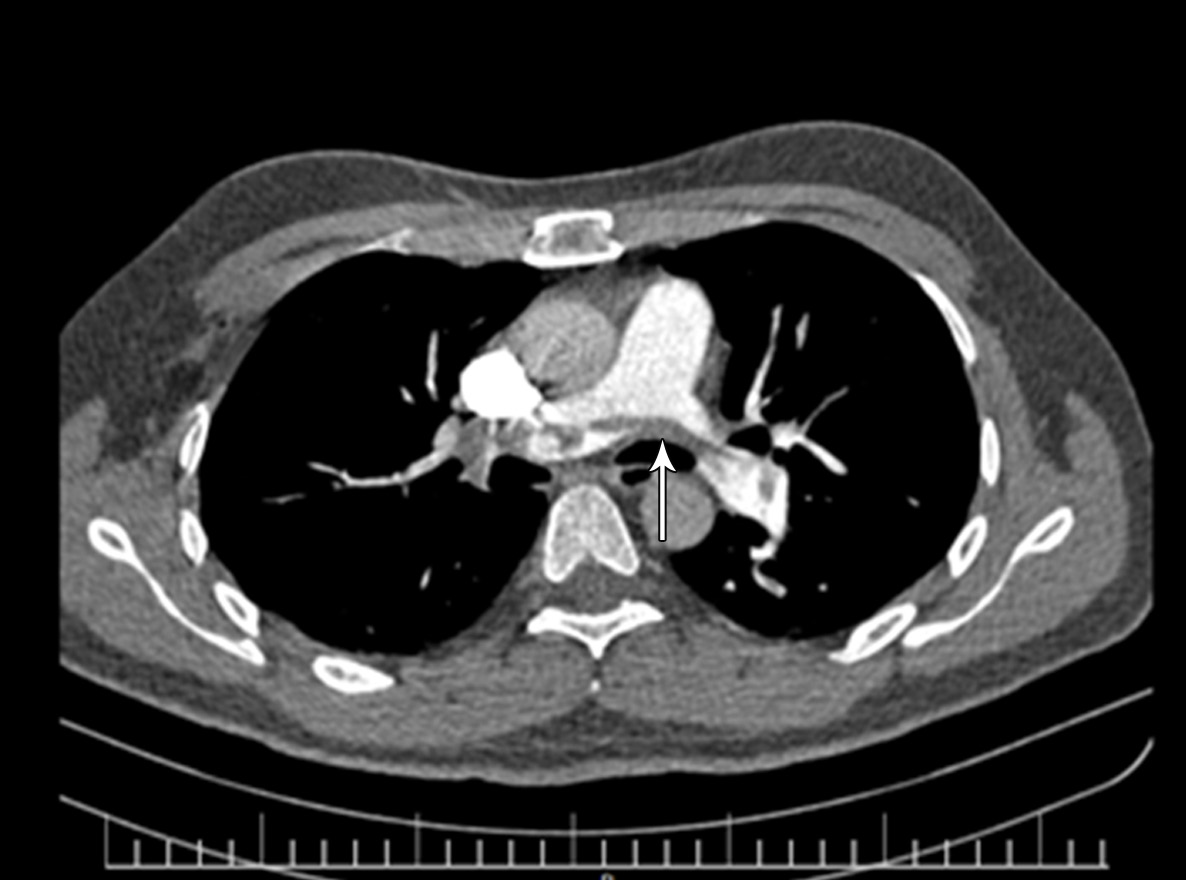
Bilateral massive pulmonary thromboembolism (arrow) in multi-detector computed tomography angiography

**Figure 3:**
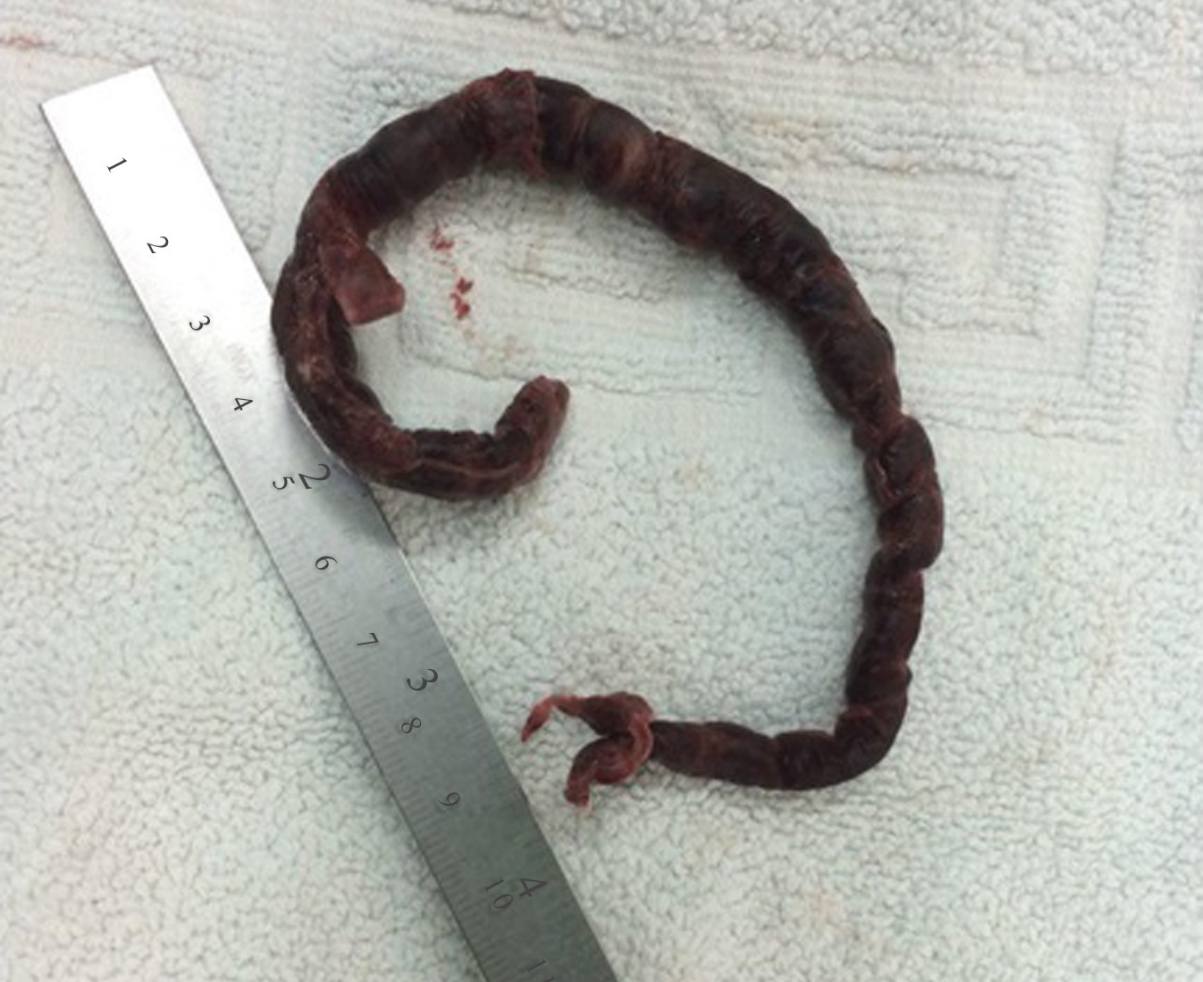
Large embolus extracted via atriotomy

## Notes:

***This paper should be cited as: ***Davarpasand T, Mohseni Badalabadi R, Mansourian S, Rahnamoun Z. Patent Foramen Ovale: A Fatal Trap. J Teh Univ Heart Ctr 2019;14(3):146-147.

## Notes:

***To watch the following videos, please refer to the relevant URLs:***

http://jthc.tums.ac.ir/index.php/jthc/article/view/911/856

Video 1. 2D transesophageal echocardiography (short-axis view) of the mid-esophageal aortic valve, showing a highly mobile mass trapped in a patent foramen ovale
